# Diverse Presentation of Secondary Aortoenteric Fistulae

**DOI:** 10.1155/2011/406730

**Published:** 2011-12-29

**Authors:** Tracey Simon, Edward Feller

**Affiliations:** Department of Health Services, Policy and Practice, Alpert Medical School, Brown University, Providence, RI 02912, USA

## Abstract

Secondary aortoenteric fistula, due to mechanical erosion or infection of a prosthetic graft, is a very rare cause of gastrointestinal bleeding and an uncommon complication of abdominal aortic aneurysm repair. A retrospective chart review conducted at our institution revealed 5 cases of secondary AEF occurring between 2006 and 2010. Presentations were diverse, including hematemesis, coffee-ground emesis, and unexplained sepsis. Delay in diagnosis was common. In reporting these cases, we seek to highlight the diverse clinical spectrum and potentially misleading features of this condition. Clinicians must retain a high index of suspicion to avoid potentially catastrophic outcomes.

## 1. Introduction

Aortoenteric fistula (AEF), due to mechanical erosion or infection of a prosthetic graft into the duodenum, is a very rare cause of gastrointestinal (GI) bleeding and an uncommon complication of abdominal aortic aneurysm (AAA) repair [1–3]. The most frequent presenting feature is upper GI (UGI) bleeding, which can range from a minor “herald” bleed to exsanguinating hemorrhage. Infrequently, AEF may manifest with atypical, nonspecific symptoms such as fever, sepsis, or unexplained abdominal pain. We report 5 cases of AEF to highlight the diverse clinical spectrum and potentially misleading features of this catastrophic condition.

## 2. Cases

Retrospective chart review at a general medical-surgical hospital over a 4-year period identified five patients, including three men and two women (age range 59–85) with AEF and a prior history of open abdominal aorta repair (Table 1). Presentations were diverse, including hematemesis, coffee-ground emesis, massive hematochezia, chronic, intermittent UGI bleeding, and unexplained sepsis. Delay in diagnosis was common. Two of three patients who underwent UGI endoscopy had initial false-negative results. One of these three had a graft visualized in the descending duodenum on repeat UGI endoscopy, performed because of rebleeding. Three patients underwent CT; however, only one was diagnostic of AEF. One patient had unsuccessful emergent, exploratory laparotomy, after cardiac arrest from exsanguinating hemorrhage. Two patients underwent successful surgical correction.

## 3. Discussion

**Figure 1:**
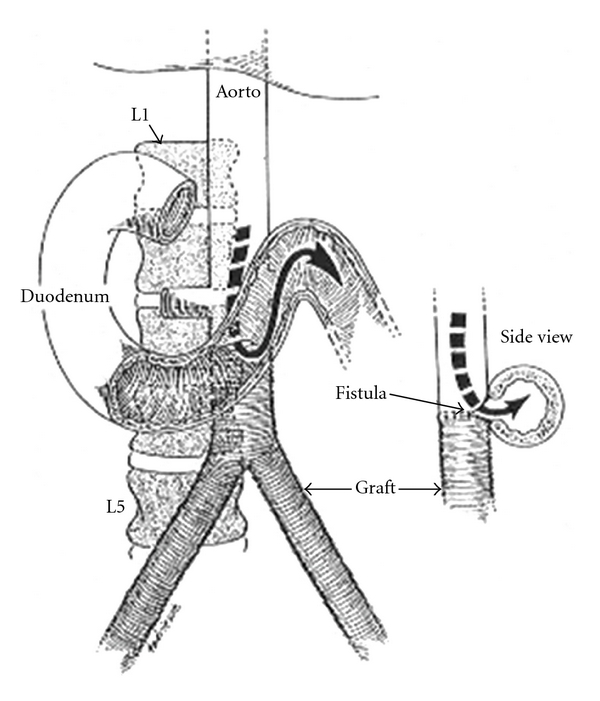
Drawing of aortoenteric fistula at the anastomosis of the abdominal aortic graft. Reproduced with permission from New England Journal of Medicine [13].

Fistulas may occur when an intact, unruptured prosthesis erodes into the duodenum or—more commonly—when the lumen of the aorta communicates directly with the bowel lumen, following graft disruption (Figure 1). Mechanisms for fistula formation include (1) bacterial seeding of a prosthetic graft, allowing extension of infection to a suture line, producing anastomotic failure. This results in perigraft infection or subsequent AEF. (2) Bowel damage, especially during emergency graft insertion, may lead to bowel wall trauma and ischemia. (3) Mechanical injury, including foreign body inflammation, trauma, devascularization, suture line failure, or graft enlargement. (4) Pseudoaneurysms or paragraft abscesses may compress, erode, or invade the bowel lumen. (5) Mechanical erosion of the GI tract may be facilitated by prosthesis-induced inflammation and adhesions.

Most AEFs are found at the 3rd or 4th portions of the duodenum, near the surgical site, but can occur in atypical locations [4, 5]. Infection is the most frequent fistula precipitant [6]. The classic, self-limited “herald bleed” may be due to mucosal bleeding from an initially intact graft or to transient occlusion of a small fistula, by clot. However, it is important to note that the classic triad of pain, UGI bleeding, and a pulsatile abdominal mass are present in less than 25% of cases [7]. Further, the self-limited nature of the “herald” bleed increases the risk that it may be missed or ignored [1, 2, 8].

**Figure 2:**
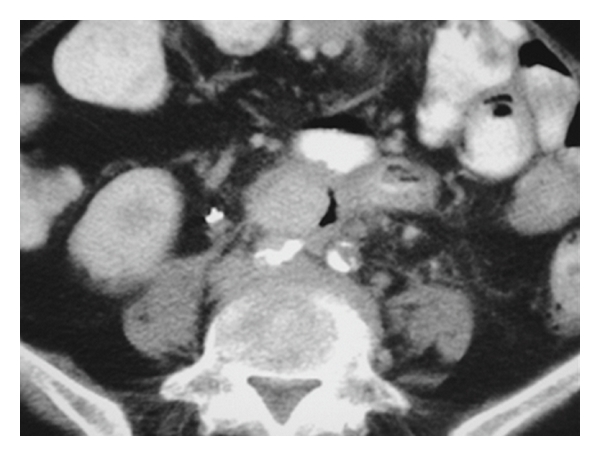
CT image of patient 5, who presented with unexplained fever. CT shows periaortic graft gas.

Sepsis may be the predominant clinical manifestation, particularly in the early stages of fistula formation, if the bowel contents remain confined to the paragraft space. In such a “paraprosthetic-enteric fistula,” the fistula tract has not yet extended into the vascular lumen. These patients will more likely present with nonspecific symptoms related to the infection itself [9]. As in one of our cases, CT imaging may reveal periaortic graft gas (Figure 2). If, over time, the infection erodes into the aorta, GI bleeding may then occur.

Fistulas present in diverse guises, including GI bleeding of any magnitude, subacute or chronic abdominal or back pain, fever, or sepsis. Unexplained fever is an uncommon and underrecognized early manifestation of AEF, as in one of our cases (Table 1). Rare and nonspecific manifestations include weight loss or malaise, lower extremity ischemia, visceral or muscle abscess, or septic arthritis due to septic emboli [5, 10]. When a patient with a prior abdominal aortic graft develops abdominal pain, GI bleeding, or sepsis, the presence of a fistula should be considered until disproved.

Delayed diagnosis is common and potentially catastrophic. A review of 18 studies indicated a median time to correct diagnosis of eight days (range: 0 hours–18 months) [10]. This group also reported a median elapsed time between the primary surgery and the occurrence of AEF of 47 months, with a range of 2 days to 24 years [10]. Possible explanations for diagnostic delay include (1) inappropriately low index of suspicion in GI bleeding; (2) GI bleeding that may be disarmingly benign; (3) occurrence of the event many years after surgery; (4) difficulty in evaluating the distal duodenum with possible false-negative result from UGI endoscopy or imaging studies; (5) atypical, nonspecific manifestations such as isolated fever or sepsis, weight loss, malaise, or abdominal pain; (6) rare occurrence without prior surgery (primary AEF from trauma, tumor, penetrating ulcer, or expanding aneurysm); (7) atypical fistula locations, such as esophagus, distal small bowel, or colon [10, 11].

Diagnostic strategy depends upon hemodynamic stability. The most frequent initial diagnostic test for possible UGI bleeding is UGI endoscopy. Visualization of a pulsatile bleeding mass or graft in the 3rd or 4th portion of the duodenum is diagnostic. AEF is strongly suggested by bleeding arising from a point distal to the 2nd portion of the duodenum—a location difficult to visualize endoscopically, with accuracy as low as 30 percent [5, 10]. Endoscopy may be most useful in detecting or excluding an alternative cause of bleeding. Use of a long, side-viewing duodenoscope may enhance fistula diagnosis by optimizing visualization of the distal duodenum [5].

CT with contrast enhancement is another useful diagnostic tool. It can demonstrate the fistula itself or detect indirect signs of infection, such as gas or liquid surrounding the graft [9, 10, 12]. CT angiography, rather than conventional angiography, is being used increasingly to visualize the aortic lumen, the aortic/graft wall, and the duodenum/jejunum, as well as to document any extravascular collections or active contrast extravasation.

In the emergent setting—common in AEF with exsanguinating bleeding—there is often insufficient time for any diagnostic procedure. Data indicates the CT scan confirms the diagnosis in only 33–80% of patients, and emergent endoscopy is rarely employed in the absence of GI bleeding [9]. Confirmation of diagnosis is therefore made by emergency surgical exploration.

## 4. Conclusion

AEF is an infrequent, life-threatening complication of aortic reconstructive surgery. Presenting features are diverse, frequently atypical, nonspecific, or disarmingly benign. Clinicians must have a high index of suspicion to avoid delayed or missed diagnoses. Endoscopists should attempt to visualize the fourth portion of the duodenum, by using a side-viewing duodenoscope, if available. If a patient with prior aortic reconstruction develops GI bleeding of any magnitude, fever, or sepsis, the presence of a fistula should be considered until disproved.

## Figures and Tables

**Table 1:** Presentation and outcomes of aortoenteric fistulae.

Case #	Presentation	Initial evaluation	Further evaluation/diagnosis	Outcome
(1) 59 M	UGI bleeding	UGI endoscopy: negative	Repeat study 2 months later showed infected graft in duodenum	Excision of infected graft; gastrojejunostomy

(2) 83 F	Fever, coffee-ground emesis, bright red blood per rectum	UGI endoscopy: negative	Episode of rebleeding prompts repeat endoscopy, which shows pulsating duodenal clot. CT reveals gas around aortic graft	Hospital death

(3) 65 M	Massive hematemesis	Emergent laparotomy	Intraoperative findings: fistula with paraaortic abscess eroding aortic and duodenal wall	Unsuccessful resuscitation

(4) 71 M	Coffee-ground emesis, normal hemoglobin	UGI endoscopy: nonbleeding graft in the 3rd portion of duodenum	Emergency laparotomy scheduled	Exsanguination en route to OR

(5) 85 M	Fever, shaking chills	Blood cultures positive for *C. albicans* and *streptococcus spp*.	CT revealed periaortic graft gas	Excision of infected graft; right aortofemoral bypass graft
